# Increased clinical frailty is associated with aortic-related mortality following fenestrated and branched endovascular repair for thoracoabdominal aortic aneurysm

**DOI:** 10.1016/j.jvs.2025.10.011

**Published:** 2025-10-14

**Authors:** Silvia Chen, Elizabeth Ramirez, Blake E. Murphy, Anjali Sribalaskandarajah, Martin Bunker, Joel Kruger, Karina A. Newhall, Rebecca A. Sorber, Sara L. Zettervall, Matthew P. Sweet

**Affiliations:** a University of Washington School of Medicine, Seattle; b Division of Vascular Surgery, University of Washington, Seattle; c Division of Vascular Surgery, University of Rochester School of Medicine and Dentistry, Rochester; d Division of Vascular Surgery, Washington University in St. Louis, St. Louis.

**Keywords:** Frailty, F/BEVAR, Clinical frailty scale, TAAA, Aortic-related mortality

## Abstract

**Objective:**

Clinical frailty is associated with reduced long-term survival after fenestrated and branched endovascular aortic repair (F/BEVAR). This study assesses the impact of phenotypic clinical frailty on perioperative outcomes and cause of death following F/BEVAR for thoracoabdominal aortic aneurysm.

**Methods:**

Patients who underwent F/BEVAR at a single institution from 2012 to 2024 were identified. The clinical frailty scale (CFS) was used to determine phenotypic frailty. Patients with a preoperative CFS of ≥4 (vulnerable) and a CFS of <4 were compared. We used χ^2^ and Fischer exact tests to compare patient demographics, anatomical and operative characteristics, and perioperative outcomes. Fine-Gray analysis was used to compare cause of death between groups. Long-term survival and reintervention were assessed with Kaplan-Meier and Cox regression analyses.

**Results:**

We included 233 patients; 60 (25.8%) had a CFS of ≥4 and 173 (74.2%) had a CFS of <4. Patients with a CFS of ≥4 were more likely to have chronic obstructive pulmonary disease (53% vs 27%) and were treated for slightly larger aneurysms (72 mm vs 68 mm; *P* = .04). There were no differences in symptomatic presentation, aneurysm extent, or operative complexity between patient groups. Additionally, there were no differences in perioperative complications including 30-day mortality, stroke, and spinal cord ischemia. Patients with a CFS of ≥4 had an increased length of hospitalization (11.3 days vs 6.9 days; *P* < .01) and were less likely to return to preoperative functional status (62.7% vs 86.1%; *P* < .01). The 3-year all-cause and aortic-related mortality rates were 35.2% and 5.7%, respectively. Patients with a CFS of ≥4 had a lower survival at 1 year (74% vs 89%), 3 years (39% vs 73%), and 5 years (25% vs 56%), compared with patients with a CFS of <4 (*P* < .01). The most common causes of death among both groups were pulmonary comorbidities (14.0%), oncologic conditions (14.0%), cardiovascular comorbidities (11.2%), and procedure-related complications (11.2%). Patients with a CFS of ≥4 were more likely to die from aortic-related mortality (10.3% vs 5.9%; *P* = .02), pulmonary comorbidities (15.4 vs 13.2%; *P* = .04), systemic decline (7.7% vs 1.5%; *P* = .02), and infection (12.8% vs 7.4%; *P* = .03). Aortic-related mortality for the entire patient cohort was 2.2% and 5.7% at 1 year and 3 years, respectively. Aortic-related deaths among clinically frail patients were often due to an inability to tolerate further aortic operations (eg, arch repair), and secondary to follow-up nonadherence in patients with a CFS of <4.

**Conclusions:**

In an expanded cohort of patients, clinical frailty was associated with lower long-term survival and an increased risk for aortic-related mortality after F/BEVAR for the treatment of thoracoabdominal aortic aneurysms. Chronic disease burden is a primary driver of overall mortality, and clinically frail patients are more likely to die from pulmonary comorbidities, infection, and systemic decline. Phenotypic frailty assessment should be considered in preoperative assessment and patient counseling before F/BEVAR.

Fenestrated and branched endovascular aortic repair (F/BEVAR) has provided a minimally invasive treatment option for thoracoabdominal aneurysm (TAAA) in patients with advanced age, complex anatomical considerations, and increased overall frailty.^1–4^ Despite ongoing advancements in endovascular techniques, device availability, and surgeon experience, long-term survival after treatment for thoracoabdominal aortic aneurysms (TAAAs) remains modest, approaching 50% at 5 years.^5,6^ Identified risk factors for this shorter survival include patient age, chronic comorbidities such as congestive heart failure, chronic obstructive pulmonary disease (COPD), renal failure, and anatomical considerations, including increased TAAA extent and aneurysm size.^6–8^ In light of this, accurate preoperative assessment and identification of high-risk patient characteristics before elective F/BEVAR remains essential to balance the competing risks of rupture vs overall clinical condition in optimizing survival benefit.

Research has demonstrated that preoperative frailty has a strong global association with increased morbidity and mortality following surgical intervention.^9,10^ With respect to vascular procedures, elevated frailty assessed using the clinical frailty scale (CFS) has demonstrated significant association with increased 30-day mortality and loss of independence after elective open and endovascular abdominal aortic aneurysm repair.^11^ A recent single-institution study from our own group evaluating a similar cohort of F/BEVAR patients demonstrated that phenotypically frail patients, that is, those patients with impaired preoperative independence and physical fitness, undergoing F/BEVAR experience impaired long-term survival.^2^ However, there is currently a paucity of data assessing outcomes and specific cause of death delineated by frailty status for this patient population. In this study, we evaluated the perioperative characteristics and cause of death stratified by CFS to identify predictors of survival and better understand the natural history of TAAA in frail patients after F/BEVAR.

## METHODS

### Study population.

This study is a single-center retrospective review of prospectively collected data from a Food and Drug Administration-approved, physician-sponsored investigational device exemption clinical trial (Identifier #NCT01874197). Patients with TAAA enrolled from 2012 to 2024 were included. This study involved treatment with physician-modified endografts (PMEGs), patient-specific commercially manufactured devices (CMDs), or an off-the-shelf thoracoabdominal endovascular graft (Zenith t-Branch, Cook Medical). All patients provided consent as part of formal enrollment in the investigational device exemption. Patients were followed postoperatively with CTA at 1 month, 6 months, and annually thereafter. Patients were grouped by a CFS of ≥4 and outcomes were compared. This study was approved by the Institutional Review Board at the University of Washington.

### CFS.

**Fig 1.**
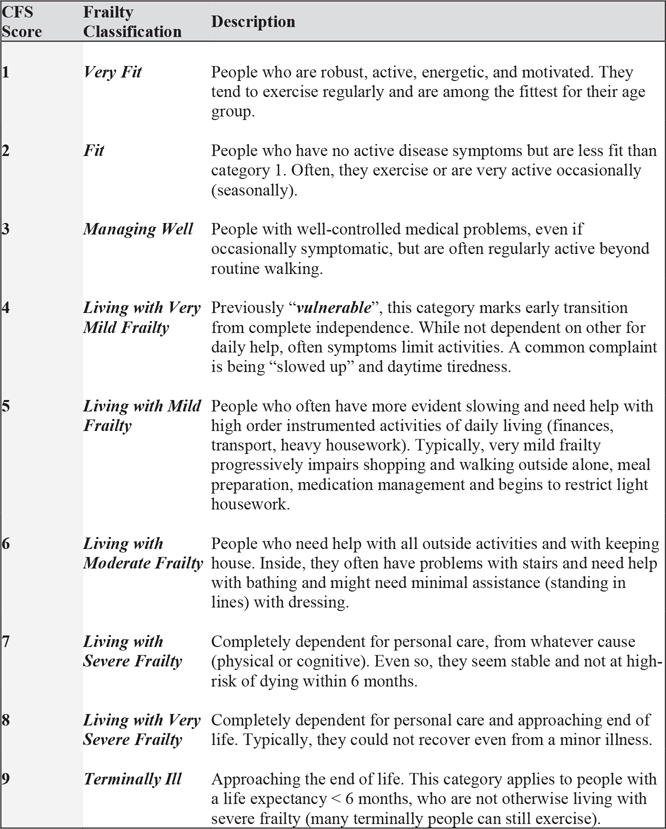
Clinical frailty scale (CFS) classification and scoring system.

The CFS is a validated and standardized tool designed to assess patient phenotypic frailty, which is a direct measurement of the patient’s functional status as assessed by their physical fitness and independence. The scale ranges from 1 (very fit) to 9 (terminally ill) based on clinical assessment and observations of physical activity, balance, and ability to independently complete activities of daily living (ADLs).^11^ A CFS of 4 correlates with vulnerability and is assigned to patients who have symptoms that limit activities but are not dependent on others for daily help (Fig 1). Clinical frailty scores were assigned preoperatively by one of two surgeon investigators (M.P.S. or S.L.Z.) as previously described^2^; inter-rater reliability was not formally assessed in this study, but multiple prior studies have demonstrated that CFS scoring between providers is highly reliable.^12,13^ This assessment included a functional status based on a focused discussion with the patient and their family to assess baseline ADLs and instrumental ADLs. Although baseline cognition was evaluated, the evaluation of functional status did not include a formal cognitive assessment.

### Demographic and comorbidities.

Demographic variables included age, sex, race, and comorbidities including diabetes mellitus, hypertension, hyperlipidemia, coronary artery disease, current or former smoking history, end-stage renal disease, a history of stroke, congestive heart failure, COPD, renal insufficiency, peripheral vascular or arterial disease, a family history of aortic dissection, and a history of connective tissue disorder. Preoperative medications including anticoagulants, antiplatelets, statins, beta-blockers, and angiotensin-converting enzyme inhibitors were compared.

### Anatomical and operative characteristics.

Anatomical characteristics included maximum aortic diameter, symptomatic presentation, aneurysm extent, prior aortic repair, and treatment for postdissection aneurysm. Operative characteristics included operative time, fluoroscopy time, fluoroscopy dose, contrast volume, estimated blood loss, prophylactic spinal drain use, device type (CMD, t-Branch, and PMEG), number of target vessels, hypogastric occlusion, and technical success. Maximum aneurysm diameter was assessed using centerline reconstruction of computed tomography angiography.

### Perioperative and long-term outcomes.

Perioperative outcomes included any major adverse event, 30-day mortality, spinal cord injury (SCI), myocardial infarction, respiratory failure, acute kidney injury (AKI) (including new dialysis), access site complications, estimated blood loss of >1 L, mesenteric ischemia, early reintervention, and intensive care unit and hospital length of stay. Respiratory failure included hypoxemic and hypercarbic respiratory failure, but excluded minor exacerbations of underlying respiratory diseases such as COPD to limit the outcome to major respiratory complications rather than adjustments in medical management (ie, administration of nebulizer treatments or short courses of medication). Temporary SCI was defined as SCI resulting in transient weakness in the lower extremities. Permanent SCI was defined as injury causing paralysis or paraparesis of the lower extremities present at the time of discharge. AKI was a composite outcome for patients with a risk of renal dysfunction, injury to the kidney, and failure of kidney function as defined by the RIFLE criteria outlined in the Society for Vascular Surgery Reporting Standards.^14^ Long-term outcomes included all-cause mortality, aortic-related mortality, and freedom from reintervention.

### Cause of death and return to preoperative baseline.

Return to preoperative functional status (RFS) was assessed between 6 months and 1 year postoperatively. RFS was determined based on clinical evaluation recorded by vascular surgery, primary care, and specialty providers within the electronic medical record after surgical intervention. Assessment of physical activity, ADLs, and instrumental ADLs relative to preoperative status, in addition to the presence of new or worsening end-stage organ dysfunction including stroke, SCI, congestive heart failure, and dialysis requirement were assessed.

In-hospital evaluation at the time of death, attending physician note, obituary search, and autopsy report when available were used to determine cause of death. Deaths were classified as aortic related if they were due to dissection, rupture, or a complication of their index procedure or secondary intervention, consistent with Society for Vascular Surgery reporting standards.^14^ Procedure-related death was defined as deaths owing to SCI at any time point in the postoperative course, or secondary to perioperative complications such as stroke, multisystem organ failure, and intracranial bleed, and any death within 30 days of index procedure. Deaths of unknown cause were not reported as aortic-related mortality if there was no evidence or symptoms suggestive of rupture, or aneurysm sac expansion in previous imaging studies. Nonaortic-related mortality included cancer, stroke, systemic decline, sepsis, trauma, gastrointestinal hemorrhage, and cardiac and pulmonary comorbidities. Cardiac causes of death included acute coronary syndrome, unstable cardiac arrhythmia, and decompensated heart failure. Pulmonary causes of death included pneumonia, acute-on-chronic respiratory failure, and perioperative respiratory failure. All patient deaths and return to functional status were adjudicated by a secondary reviewer.

### Statistical analysis.

Univariate analysis of demographics, comorbidities, anatomical, and operative variations was performed using Pearson χ^2^ or Fischer exact test for nominal data. The Student *t* test and Mann-Whitney *U* test were used for the analysis of continuous variables based on normality of distribution. Perioperative and late outcomes were determined using logistic regression analysis for binomial outcomes and linear regression analysis for continuous outcomes. Cause of death outcomes were determined using Fine and Gray methods. All-cause mortality, reintervention, and aortic-related mortality were assessed with Kaplan-Meier survival analysis. Cox regression analysis was completed for all-cause mortality and reintervention with adjustment for maximum aneurysm diameter, COPD, congestive heart failure, symptomatic presentation, postdissection aneurysm, preoperative CKD stage 4 or 5 and aneurysm extent. These variables were selected for inclusion as those with statistically significant differences on univariate analysis in the present study or those with known association with mortality and reintervention based on prior published studies. *P* values of <.05 were considered statistically significant. All statistical analyses were conducted in Python or R Studio. No variables had >10% missing data.

## RESULTS

### Study population.

A total of 233 patients underwent F/BEVAR from 2012 to 2024―60 patients (25.8%) had a CFS of ≥4 and 173 (74.2%) had a CFS of <4. The patient cohort was predominantly male (72.5%) and White (92.3%). COPD was more common in patients with a CFS of ≥4 (53.3% vs 27.2%; *P* < .01) (Table I). There were no other differences demographics, comorbidities, and preoperative medications. Patients with a CFS of ≥4 had a slightly larger maximum aneurysm size diameter (72 ± 14 mm vs 68 ± 11 mm; *P* = .04), although there were no differences in aneurysm extent, symptomatic presentation, history of prior aortic repair, or treatment for postdissection aneurysm between the patient groups (Table II). Additionally, there were no differences in operative details including device type, number of target vessels, operative time, total fluoroscopy time, contrast material use, estimated blood loss, hypogastric occlusion, prophylactic lumbar drain, or technical success rates (Table II).

### Perioperative outcomes.

A total of nine patients (3.9%) died within 30 days of index procedure, including seven patients (4.0%) with a CFS of <4 and two patients (3.3%) with a CFS of ≥4 (*P* = .36). Importantly, for patients with a CFS of ≥4, there were no differences in major adverse events (31.7% vs 22%; *P* = .14), SCI (16.7% vs 8.7%; *P* = .09), stroke (1.7% vs 5.2%; *P* = .27), or AKI (3.3% vs 3.5%) compared with patients with a CFS of <4. However, hospital length of stay was longer for patients with a CFS of ≥4 (11 ± 12 days vs 7 ± 7 days; *P* < .01) (Table III).

### Long-term outcomes.

**Fig 2.**
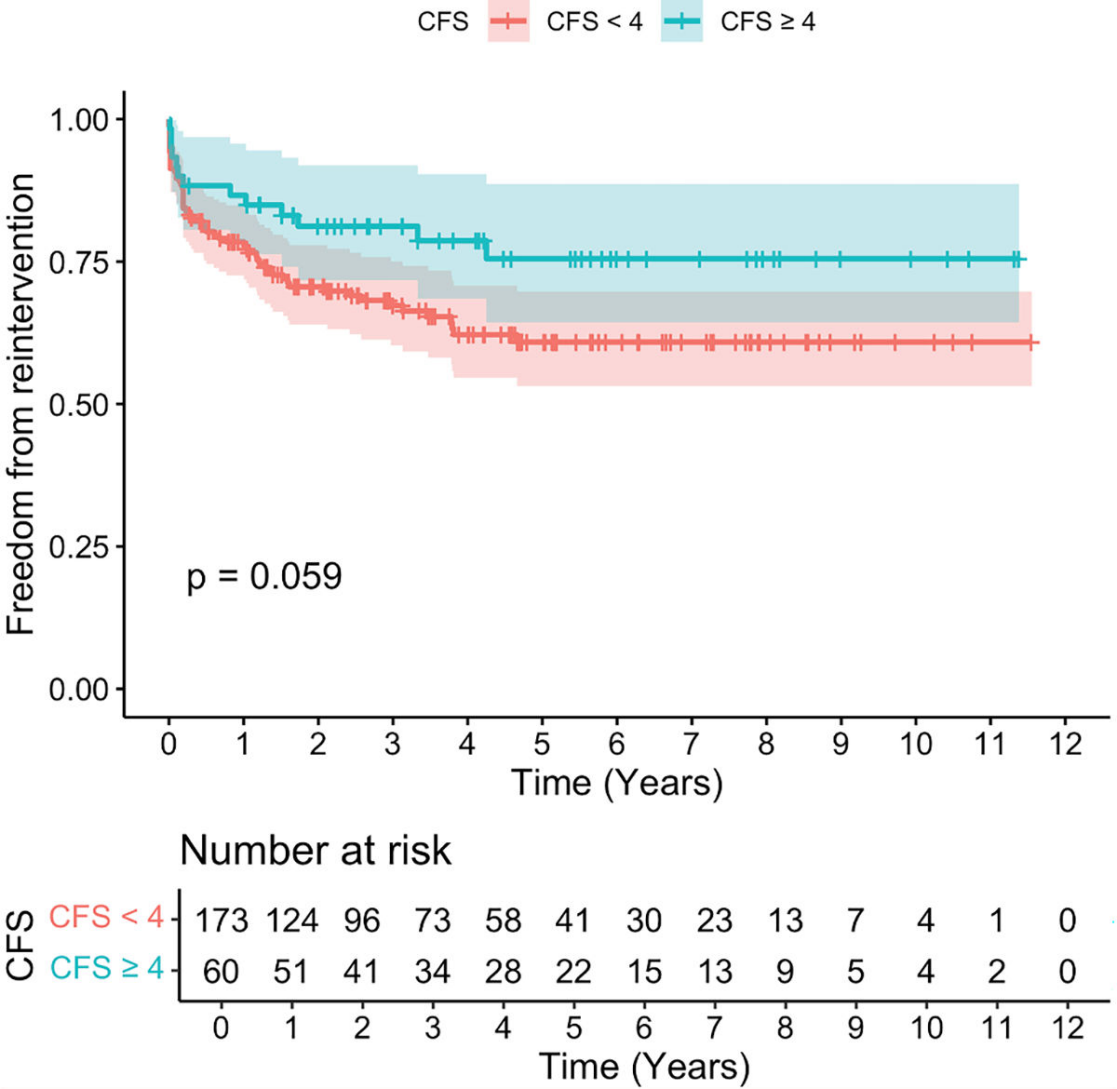
Kaplan-Meier analysis for freedom from reintervention based on clinical frailty status (clinical frailty score [CFS]).

Of the 233 patients who underwent F/BEVAR, a total of 72 (30.9%) underwent reintervention, including 59 patients (34.1%) with a CFS of <4 and 13 patients (21.7%) with a CFS of ≥4 (Table IV). There were no differences in freedom from reintervention at 1 year (86.6% vs 77.8%), 3 years (81.2% vs 67.3%), or 5 years (75.5% vs 60.9%) between patients with a CFS of ≥4 and a CFS of <4, respectively (Fig 2) (*P* = .059). On adjusted analysis, only postdissection aneurysm was associated with increased risk for reintervention (hazard ratio [HR], 2.41; 95% confidence interval [CI], 1.34–4.31) (Table V). After F/BEVAR, a total of 224 patients, including 165 patients with a CFS of <4 and 59 patients with a CFS of ≥4, had complete data available to assess RFS. Of note, patients with a CFS of ≥4 were less likely to RFS (62.7% vs 86.1%; *P* < .01) compared with those with a CFS of <4 (Table IV).

### Cause of death.

**Fig 3.**
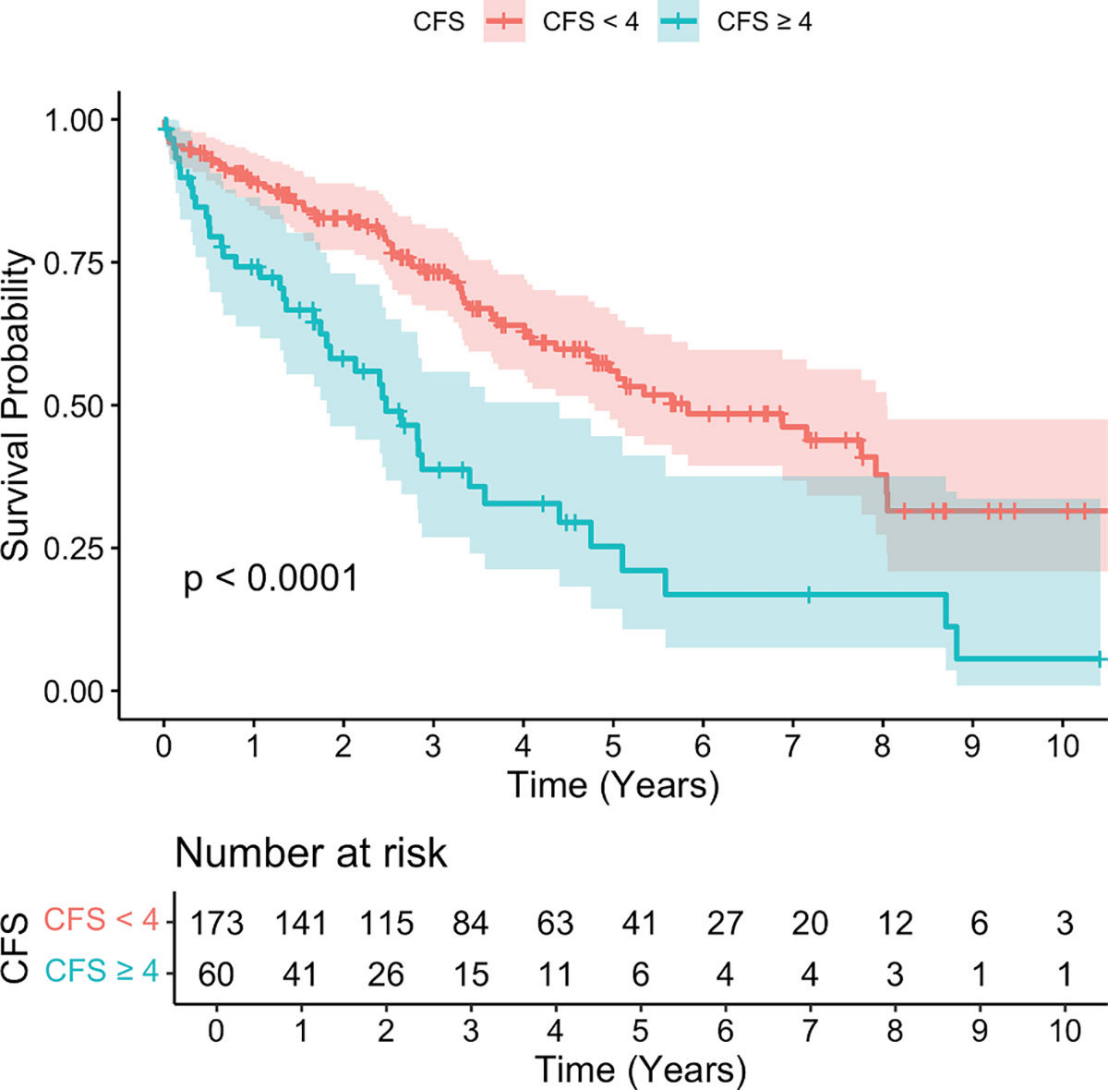
Kaplan-Meier survival analysis for all-cause mortality based on clinical frailty status (clinical frailty score [*CFS*]).

Overall survival was 85.5% at 1 year post F/BEVAR, 64.8% at 3 years, and 48.5% at 5 years. Patients with a CFS of ≥4 had reduced survival at 1 year (74.2% vs 89.4%), 3 years (38.8% vs 73.4%), and 5 years (25.3% vs 56%), compared with patients with a CFS of <4 (Fig 3; *P* < .01). Of the 233 patients who underwent F/BEVAR, a total of 107 patients (45.9%) died, including 39 patients (63.5%) with a CFS of ≥4 and 68 patients (36.4%) with a CFS of <4 (Table IV). On adjusted analysis, a CFS of ≥4 (HR, 1.91; 95% CI, 1.24–2.93), CKD stage IV or V (HR, 2.37; 95% CI, 1.18–4.79), and COPD (HR, 1.58; 95% CI, 1.05–2.37) were associated with increased mortality (Table VI).

The most common causes of death among both groups were related to pulmonary comorbidities (14.0%), oncologic conditions (14.0%), cardiovascular comorbidities (11.2%), and procedure-related complications (11.2%). However, patients with a CFS of ≥4 were more likely to die from aortic-related causes (10.3% vs 5.9%; *P* = .02), pulmonary causes (15.4 vs 13.2%; *P* = .04), systemic decline (7.7% vs 1.5%; *P* = .02), and infection (12.8% vs 7.4%; *P* = .03). There were no differences in procedure-related, gastrointestinal, neurological, or trauma-related complications, cardiovascular comorbidities, oncologic conditions, renal failure, and unknown causes of death based on clinical frailty status (Table III).

### Aortic-related mortality.

**Fig 4.**
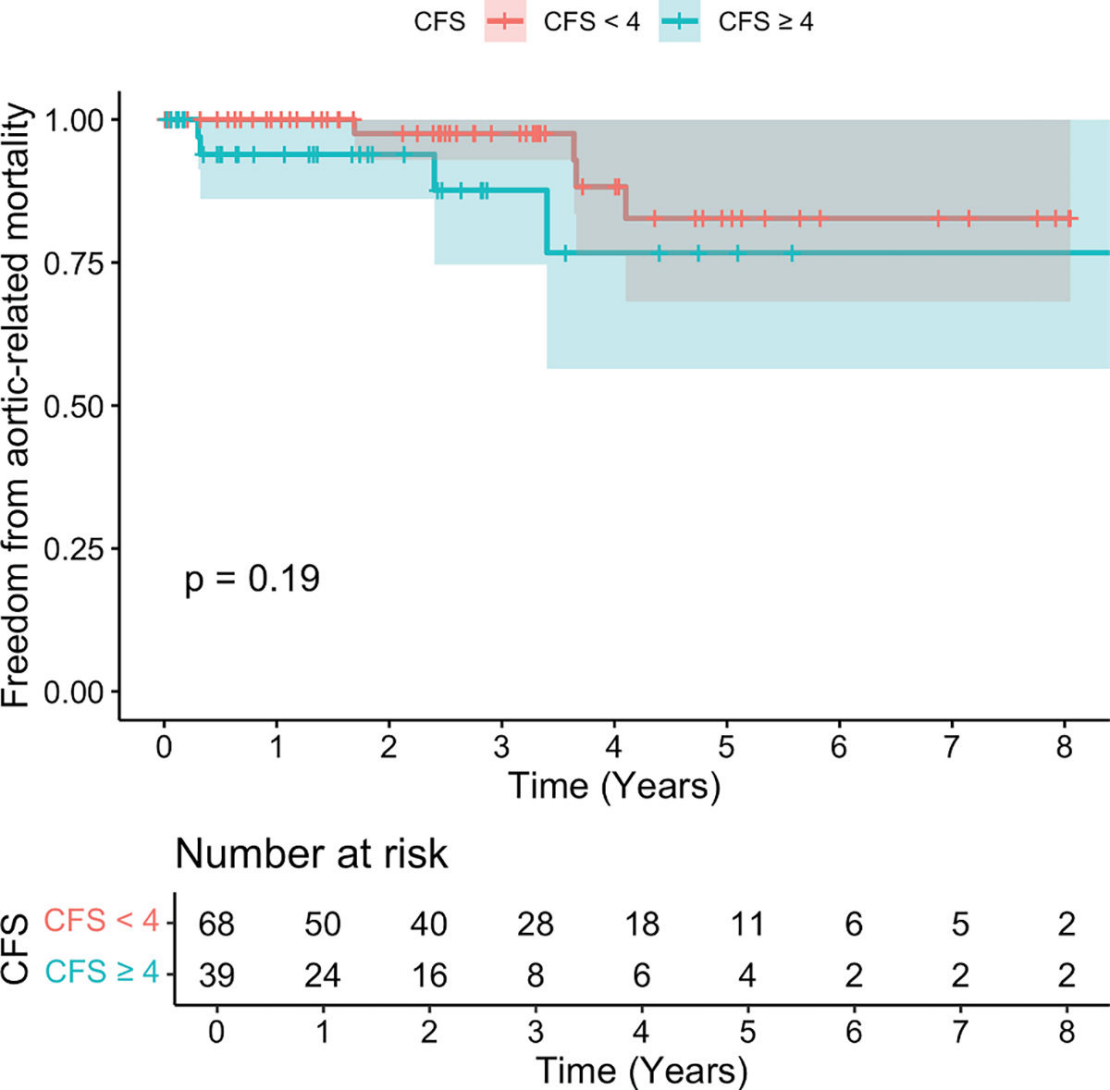
Kaplan-Meier survival analysis for aortic-related mortality based on clinical frailty status (clinical frailty score [*CFS*]).

Overall freedom from aortic-related mortality for the entire patient cohort was 97.8% at 1 year and 94.3% at 3 years. In time-to-event analyses, there were no differences in freedom from aortic-related mortality for patients with a CFS of ≥4 and a CFS of <4 at 1 year (93.9% vs 100%), 3 years (87.7% vs 97.6%), of 5 years (76.7% vs 82.8%) (Fig 4) (*P* = .19). Given study power, an adjusted analysis was not performed.

Of the eight patients who died secondary to aortic-related mortality, four patients (50%) had a CFS of ≥4. Two clinically frail patients with extent II TAAAs died of complications related to arch interventions for treatment of proximal aortic degeneration after a three-vessel fenestrated PMEG and four-vessel branched CMD, respectively. The third patient, who initially underwent zone 0 TBE for treatment of an arch aneurysm, presented with acute respiratory failure, sepsis, and concern for an aortobronchial fistula 6 months after tbranch for treatment of an extent I TAAA. The fourth patient died secondary to ongoing sac expansion and subsequent rupture from a known arch aneurysm 2.5 years after their index four-vessel fenestrated PMEG for treatment of an extent I TAAA. The patient declined redo sternotomy and opted for comfort measures at the time of rupture owing to their advanced age and progressively worsening clinical frailty.

Among patients with a CFS of <4, one patient experienced rupture secondary to rapid aneurysm expansion to 14 cm 3 years after four-fenestrated PMEG for an extent II TAAA. They had been previously lost to follow-up within 6 months of their index intervention. A second patient died 3 years after treatment of an extent IV TAAA with four-vessel fenestrated PMEG owing to rupture of a type Ia endoleak. Before their death event, the patient had refused reintervention for a known type IIIc endoleak identified on surveillance imaging. A third patient experienced recurrent rupture after prior TEVAR extension and visceral vessel relining of a four-vessel fenestrated CMD for treatment of an extent III TAAA. Despite ongoing sac expansion, reintervention was not offered, given the lack of identifiable endoleak on repeat imaging and worsening clinical status. The final patient died 2 years after three-vessel fenestrated CMD for treatment of an extent I TAAA. Despite undergoing zone 2 arch replacement 6 months prior for proximal aortic degeneration and rigorous postoperative surveillance, the patient experienced ongoing sac expansion and subsequent rupture.

## DISCUSSION

This study provides important follow-up to prior research demonstrating lower long-term survival for clinically frail patients undergoing F/BEVAR for TAAAs.^2^ This expanded analysis includes a larger cohort of patients with assessment of comorbidities, perioperative outcomes, survival, and cause of death based on phenotypic clinical frailty. Although there were few differences in the overall comorbidity profile or perioperative complications, clinically frail patients demonstrated an increased absolute risk of aortic-related mortality. Cause of death analysis revealed that chronic disease burden was the primary driver of all-cause mortality for the entire patient cohort; however, when adjusting for comorbidities, a higher CFS remained an independent predictor of overall mortality. To the authors’ knowledge, this study provides a novel assessment of aortic-related mortality by phenotypic frailty status for patients after F/BEVAR.

Aortic-related mortality accounted for 7.5% of all deaths (8 patients) in the study cohort, with statistically significant differences on univariate analysis between those with a CFS of ≥4 (10.3%) and a CFS of <4 (5.9%; *P* < .02). Although this study was underpowered to demonstrate a time-dependent difference in aortic-related mortality stratified by CFS (*P* = .19), overall aortic-related mortality was 2.2% and 5.7% at 1 year and 3 years, respectively. These findings mirror outcomes from two recent multi-institutional studies of F/BEVAR reporting aortic-related mortality between 3.8% and 10.9%.^5,6^ Although patients with a CFS of ≥4 had slightly larger maximum aneurysm diameters, older age, and greater aneurysm extent―previously identified predictors of aortic-related mortality^5^ ―were similar between patient groups. Although there were no differences in other risk factors, such as perioperative complications or reintervention, clinically frail patients were twice as likely to have COPD. COPD was associated with allcause mortality after F/BEVAR in the current study and is historically associated with aortic rupture in patients treated for AAA.^15^ However, all clinically frail patients with aortic-related mortality were nonsmokers or remote former smokers, and only one individual carried a preoperative COPD diagnosis. It is important to recognize that all clinically frail patients with aortic-related mortality experienced proximal disease progression with 75% of deaths related to mid-term arch and thoracic reinterventions. Although this study was not powered for adjusted analysis, these data suggest that clinical frailty may be a novel preoperative risk factor for aortic-related death.

In addition to differences regarding aortic-related mortality, clinically frail patients were more likely to die from pulmonary comorbidities (15.4% vs 13.2%), infection (12.8% vs 7.4%), and systemic decline (7.7% vs 1.5%). The greater prevalence of COPD may account for increased risk for death from pulmonary causes in clinically frail patients. Meanwhile, the higher rates of mortality secondary to infectious insults and systemic decline for this cohort is likely explained by the diminished physiological reserve and subsequent vulnerability for developing increased dependency when exposed to a stressor for clinically frail patients.^16,17^ Last, the current study redemonstrated increased all-cause mortality for clinically frail patients at 1 year, 3 years, and 5 years postoperatively. That all four frail patients experiencing aortic-related mortality died owing to complications related to or an inability to tolerate an arch procedure is important to note. Although the adjusted model suggests that CFS, not aneurysm extent, is a predictor of overall mortality, aneurysm extent remains an important consideration when counseling patients with regards to medium-to long-term outcomes. These data suggest that, even if frail patients are able to tolerate F/BEVAR, they may not be able to tolerate a normal spectrum of reinterventions, the morbidity of which are increased with patients with more extensive aneurysms, particularly those involving the aortic arch.

Importantly, cause of death analysis revealed that respiratory disease (14.0%), cancer (14.0%), and cardiovascular complications (11.2%) were the leading causes of death for the entire study cohort. Ten individuals had an unknown cause of death (9.3%) with complete mortality data available for 91% of patients. Multi-institutional studies have previously found that cardiac, respiratory, and infection-related complications were the primary cause of death for patients with juxtarenal AAA and TAAA treated with PMEG.^6^ Meanwhile, cardiovascular disease was identified as the most frequent cause of death, accounting for 33% of all mortality after F/BEVAR for TAAA.^5^ Notably, these studies had higher rates of missing data at 40% and 17%, respectively. These collective data underscore that chronic disease burden cannot be underestimated in this complex patient population, even after successful treatment for aortic-related pathology.

There are other important clinical considerations and implications in the current study. Although there were no significant differences based on clinical frailty status, 16.7% of patients with a CFS of ≥4 experienced SCI compared with 8.7% of those with a CFS of <4. Risk factors for SCI after F/BEVAR including advanced age, peripheral vascular disease, postdissection aneurysm, and extent I, II, and III aneurysms^18–20^ ―which were comparable between patient cohorts. Although likely underpowered, the current study findings may signal a potential association between clinical frailty and SCI after F/BEVAR, which warrants future investigation. Additionally, two patients with a CFS of <4 who experienced aortic-related mortality refused elective reintervention or were lost to clinical follow-up, highlighting the importance of appropriate postoperative surveillance after complex aortic repair to identify individuals who would benefit from minor, elective reintervention and prevent aortic-related morbidity, as it is known from prior research that minor reinterventions following F/BEVAR are associated with improved overall long-term survival.^21^

Given the collective risk for reduced postoperative functional status and long-term survival and increased aortic-related mortality, a higher aneurysm size threshold for repair may be considered for clinically frail patients. The clinically modest though statistically significant increase in aneurysm size in the CFS ≥4 group likely reflects that this clinical decision-making is already occurring on the part of the providers. Existing clinical practice guidelines for repair threshold in the context of isolated AAA suggest the annual risk of rupture for a 5.5- to 6.0-cm AAA is 2.2% to 5.4%, which increases to 3.2% to 6.4% for 6.0 to 7.0 cm and 5.2% to 7.9% for aneurysms >7.0 cm.^22^ These data cannot be perfectly extrapolated to TAAA, because it is known that there is a dramatic inflection point in rupture risk for this disease process at 6.0 cm,^23^ increasing to 10% to 14% at 6.5 cm,^24,25^ with a secondary dramatic inflection point at 6.5 cm to 30% to 40%.^25^ Although risk assessment must be individualized, these data suggest that a larger size threshold than the standard 6.0 cm be used for repair of asymptomatic TAAA among frail patients. The present data are being used to inform the preoperative counseling process for F/BEVAR at our own institution.

This study was performed at a single institution with strict inclusion and exclusion criteria and thus may not be universally generalizable to the care of all patients with TAAA, particularly those with less extensive TAAA. Although the current study represents a large sample size for a single institution, differences may exist which could not be identified owing to inadequate study power―particularly spinal cord ischemia, differences between major and minor reinterventions, and adjusted and time-dependent analyses for reintervention and aortic-related mortality. There were 10 patients with an unknown cause of death, accounting for 9.3% of the patient cohort that died. It should be noted that extensive chart biopsy, obituary search, and direct contact was performed to confirm patient vital status. The retrospective nature of this study precluded the collection of qualitative data surrounding the potential for reduced quality of life; however, this represents a future opportunity for assessment of patient-reported outcomes regarding the postoperative impact of complex aneurysm repair for frail patients.

## CONCLUSIONS

Phenotypically frail patients have reduced long-term survival and increased risk for aortic-related mortality following F/BEVAR for treatment of TAAA. Chronic disease burden is a primary driver of overall mortality, whereas clinically frail patients are more likely to die from pulmonary comorbidities, infection, and systemic decline. These data suggest that clinical frailty status may be a novel preoperative risk factor for aortic-related mortality. Phenotypic frailty assessment should be considered in preoperative risk stratification and patient counseling before F/BEVAR.

## Figures and Tables

**Table I.** Demographics and comorbidities for patients based on clinical frailty status

		CFS		
			
	All patients (n = 233)	<4 (n = 173)	≥4 (n = 60)	*P* value

Age, years	72.8 ± 7.2	72.5 ± 7.4	73.7 ± 6.8	.26
Male sex	169 (72.5)	127 (73.4)	42 (70.0)	.73
Race				
Native American	2 (0.9)	2 (1.2)	0 (0)	1.00
Black or African American	6 (2.6)	3 (1.7)	3 (5.0)	.18
White	215 (92.3)	160 (92.5)	55 (91.7)	1.00
Asian	4 (1.7)	4 (2.3)	0 (0)	.57
Other	6 (2.6)	4 (2.3)	2 (3.3)	.65
Comorbidities				
Stroke	27 (11.6)	18 (10.4)	9 (15.0)	.52
Coronary artery disease	103 (44.2)	75 (43.4)	28 (46.7)	.72
Hyperlipidemia	166 (71.2)	119 (68.8)	47 (78.3)	.24
Hypertension	209 (89.7)	152 (87.9)	57 (95.0)	.21
Congestive heart failure	23 (9.9)	17 (9.8)	6 (10.0)	1.00
CABG	43 (18.5)	31 (17.9)	12 (20.0)	.88
COPD	79 (33.9)	47 (27.2)	32 (53.3)	**<.01**
Smoking history	197 (84.5)	146 (84.4)	51 (85.0)	1.00
Diabetes	29 (12.4)	25 (14.5)	4 (6.7)	.17
Renal insufficiency	11 (4.7)	6 (3.5)	5 (8.3)	.24
PVD/PAD	40 (17.2)	28 (16.2)	12 (20.0)	.65
Family history of aortic dissection	48 (20.6)	36 (20.8)	12 (20.0)	1.00
History of connective tissue disorder	4 (1.7)	3 (1.7)	1 (1.7)	1.00
Preoperative medications				
Anticoagulants	43 (18.5)	29 (16.8)	14 (23.3)	.36
Antiplatelets	17 (7.3)	12 (6.9)	5 (8.3)	.95
Statins	176 (75.5)	131 (75.7)	45 (75.0)	1.00
Beta-blockers	139 (59.7)	104 (60.1)	35 (58.3)	.89
ACE inhibitors	105 (45.1)	83 (48.0)	22 (36.7)	.15

*ACE*, Angiotensin-converting enzyme; *CABG*, coronary artery bypass graft; *CFS*, clinical frailty scale; *COPD*, chronic obstructive pulmonary disease; *PVD/PAD*, peripheral vascular disease/peripheral arterial disease.

COPD is defined as use of home O_2_ or COPD exacerbation leading to hospitalization.

Values are mean ± standard deviation or number (%).

Boldface entries indicate statistical significance.

**Table II.** Anatomical and operative characteristics based on clinical frailty status

		CFS		
			
	All patients (n = 233)	<4 (n = 173)	≥4 (n = 60)	*P* value

Anatomical characteristics				
Maximum aneurysm diameter	68.7 ± 11.8	67.6 ± 10.7	71.8 ± 14.1	**.04**
Symptomatic presentation	25 (10.7)	15 (8.7)	10 (16.7)	.15
Extent I-III TAAA	130 (55.8)	95 (54.9)	35 (58.3)	.76
Extent IV-V TAAA	90 (38.6)	70 (40.5)	20 (33.3)	.41
Suprarenal/pararenal	13 (5.6)	8 (4.6)	5 (8.3)	.45
Prior aortic repair	125 (53.6)	89 (51.4)	36 (60.0)	.34
Postdissection aneurysm	28 (12.0)	24 (13.9)	4 (6.7)	.17
Operative details				
Operative time, minutes	263.7 ± 96.9	264.8 ± 100.6	260.6 ± 86.0	.76
Total fluoroscopy time, minutes	56.8 ± 23.4	57.7 ± 24.7	54.2 ± 18.9	.26
Contrast material use, mL	151.1 ± 43.8	151.7 ± 43.3	149.4 ± 45.6	.74
Estimated blood loss, mL	214.5 ± 266.7	223.5 ± 292.4	188.5 ± 171.5	.27
Prophylactic lumbar drain	99 (42.5)	76 (43.9)	23 (38.3)	.55
No. of target vessels	3.9 ± 0.5	3.9 ± 0.5	3.9 ± 0.5	.75
Device type				
t-branch	54 (23.2)	37 (21.4)	17 (28.3)	.32
CMD	126 (54.1)	98 (56.6)	28 (46.7)	.28
PMEG	52 (22.3)	38 (22.0)	14 (23.3)	.92
Hypogastric occlusion	29 (12.4)	20 (11.6)	9 (15.0)	.65
Technical success	217 (93.1)	160 (92.5)	57 (95.0)	.77

*CFS*, Clinical frailty scale; *CMD*, custom-manufactured device; *PMEG*, physician-modified endograft; *TAAA*, thoracoabdominal aneurysm.

Values are mean ± standard deviation or number (%). Boldface entries indicate statistical significance.

**Table III.** Perioperative outcomes based on clinical frailty status

		CFS		
			
	All patients (n = 233)	<4 (n = 173)	≥4 (n = 60)	*P* value

30-Day mortality	9 (3.9)	7 (4.0)	2 (3.3)	.36
Any 30-day major adverse event	57 (24.5)	38 (22.0)	19 (31.7)	.14
Early reintervention	20 (8.6)	16 (9.2)	4 (6.7)	.54
Spinal cord ischemia	25 (10.7)	15 (8.7)	10 (16.7)	.09
Permanent	12 (5.2)	8 (4.6)	4 (6.7)	.54
Temporary	13 (5.6)	7 (4.0)	6 (10.0)	.09
Myocardial infarction	12 (5.2)	9 (5.2)	3 (5.0)	.95
Stroke	10 (4.3)	9 (5.2)	1 (1.7)	.27
Respiratory failure	24 (10.3)	15 (8.7)	9 (15.0)	.17
AKI	8 (3.4)	6 (3.5)	2 (3.3)	.96
Mesenteric ischemia	1 (0.4)	1 (0.6)	0 (0)	N/A
Access site complication	3 (1.3)	2 (1.2)	1 (1.7)	.76
EBL > 1000 mL	2 (0.9)	2 (1.2)	0 (0)	N/A
Hospital stay length, days	8 ± 8.7	6.9 ± 6.9	11.3 ± 12.2	**<.01**
ICU stay length, days	4.2 ± 5.7	3.8 ± 4.8	5.5 ± 7.8	.07

*AKI*, Acute kidney injury; *CFS*, clinical frailty scale; *EBL*, estimated blood loss; *ICU*, intensive care unit.

Values are number (%) or mean ± standard deviation.

Boldface entries indicate statistical significance.

Note: Early reintervention is any reintervention performed within 30 days of initial procedure.

**Table IV.** Late outcomes and cause of death based on clinical frailty status

		CFS		
			
	All patients (n = 233)	<4 (n = 173)	≥4 (n = 60)	*P* value

Return to preoperative baseline^a^	179 (79.9)	142 (86.1)	37 (62.7)	**<.01**
Reintervention at any point	72 (30.9)	59 (34.1)	13 (21.7)	.08
Cause of death	(n = 107)	(n = 68)	(n = 39)	
Aortic related	8 (7.5%)	4 (5.9%)	4 (10.3%)	**.02**
Procedure related	12 (11.2)	7 (10.3)	5 (12.8)	.19
Cardiovascular	12 (11.2)	9 (13.2)	3 (7.7)	.56
Oncologic	15 (14.0)	10 (14.7)	5 (12.8)	.12
Neurological	6 (5.6)	6 (8.8)	0 (0)	N/A
Gastrointestinal	4 (3.7)	4 (5.9)	0 (0)	N/A
Infectious	10 (9.4%)	5 (7.4)	5 (12.8)	**.03**
Systemic decline	4 (3.7)	1 (1.5)	3 (7.7)	**.02**
Trauma related	6 (5.6)	3 (4.4)	3 (7.7)	.08
Renal failure	2 (1.9)	1 (1.5)	1 (2.6)	.33
Pulmonary	15 (14.0)	9 (13.2)	6 (15.4)	**.04**
Other	3 (2.8)	2 (2.9)	1 (2.6)	.52
Unknown cause of death	10 (9.3)	7 (10.3)	3 (7.7)	.42

*CFS*, Clinical frailty scale.

Values are number (%). Boldface entries indicate statistical significance.

a Denominator based on number of patients with 1-year of postoperative data available (165 patients for CFS of <4 and 59 patients with CFS of ≥4).

**Table V.** Adjusted analysis for reintervention

	HR	95% CI

CFS ≥4	0.65	0.35–1.22
Maximum aneurysm diameter	0.99	0.97–1.01
COPD	1.02	0.59–1.76
Congestive heart failure	0.44	0.14–1.42
History of aortic dissection	**2.27**	**1.21**–**4.27**
Chronic kidney disease stage IV or V	0.62	0.15–2.58
Symptomatic presentation	0.52	0.18–1.46
Extent I-III aneurysm	0.42	0.16–1.12
Extent IV-V aneurysm	0.31	0.11–0.84

*CFS*, Clinical frailty scale; *CI*, confidence interval; *COPD*, chronic obstructive pulmonary disease; *HR*, hazard ratio.

Boldface entries indicate statistical significance.

**Table VI.** Adjusted analysis for long-term survival

	HR	95% CI

CFS ≥ 4	**1.91**	**1.24, 2.93**
Maximum aneurysm diameter	1.02	1.00, 1.03
COPD	**1.58**	**1.05, 2.37**
Congestive heart failure	1.00	0.46, 2.19
History of aortic dissection	1.04	0.53, 2.05
Chronic kidney disease stage IV or V	**2.36**	**1.16, 4.78**
Symptomatic presentation	1.56	0.88, 2.73
Extent I-III aneurysm	1.56	0.37, 6.56
Extent IV-V aneurysm	1.42	0.34, 6.00

*CFS*, Clinical frailty scale; *COPD*, chronic obstructive pulmonary disease; *HR*, hazard ratio.

## ARTICLE HIGHLIGHTS

- **Type of Research:** Single-center retrospective review of prospectively collected data from a physician-sponsored investigational device exemption clinical trial
- **Key Findings:** We included 233 patients underwent fenestrated and branched endovascular aortic repair for the treatment of thoracoabdominal aortic aneurysm from 2012 to 2024. Phenotypic frailty was assessed using the clinical frailty scale (CFS) and patients were stratified by clinical CFS <4 and ≥4. Overall survival for patients with a CFS of ≥4 was lower at 1 year (74% vs 89%), 3 years (39% vs 73%), and 5 years (25% vs 56%), compared with patients with a CFS of <4. The most common causes of death among both groups were pulmonary comorbidities (14.0%), oncologic conditions (14.0%), cardiovascular comorbidities (11.2%), and procedure-related complications (11.2%). Patients with a CFS of ≥4 died more frequently from aortic-related mortality (10% vs 6%; *P* = .02). Aortic-related mortality for the entire patient cohort was 2.2% and 5.7% at 1 year and 3 years, respectively.
- **Take Home Message:** Approximately one-half of patients who undergo fenestrated and branched endovascular aortic repair for the treatment of thoracoabdominal aneurysm die from chronic medical comorbidities within 5 years of repair. However, patients with a CFS of ≥4 have significantly lower long-term survival and increased risk for aortic-related mortality.
